# Community Psychology as a Process of Citizen Participation in Health Policy: Comment on "The Rise of Post-truth Populism in Pluralist Liberal Democracies: Challenges for Health Policy"

**DOI:** 10.15171/ijhpm.2017.72

**Published:** 2017-06-25

**Authors:** Danny Taggart

**Affiliations:** School of Health and Human Sciences, University of Essex, Colchester, UK.

**Keywords:** Populism, Patient and Public Involvement (PPI), Community Psychology, Participation

## Abstract

This brief commentary discusses a recent paper by Speed and Mannion that explores "The Rise of post truth populism in liberal democracies: challenges for health policy." It considers their assertion that through meaningful democratic engagement in health policy, some of the risks brought about by an exclusionary populist politics can be mediated. With an overview of what participation means in modern healthcare policy and implementation, the field of community psychology is presented as one way to engage marginalized groups at risk of exploitation or exclusion by nativist populist policy.

In the ‘Rise of post-truth populism in liberal democracies: challenges for health policy,’^1^ citizen participation is proffered as one antidote to the post-truth assertions of populist political movements such as the election in the United States of Donald Trump and the vote for Brexit in the United Kingdom. A paradox is noted whereby on the one hand populism which is often characterized by a nativist policy agenda offers the promise of increased democratic accountability by taking power away from unelected elites; exemplified by the sound bites of “draining the swamp” in the United States and “people have had enough of experts” in the United Kingdom. On the other hand, populism conceived this way does not deliver the promised power back to the people but rather populist leaders utilize social media and other sites of engagement to promote ‘fake news’ and ‘alternative facts’ in order to move into a ‘post-truth’ political landscape where the populace can be manipulated and controlled through a range of discursive techniques. Speed and Mannion^1^ argue that this is relevant for the health of populations and the implementation of healthcare policy in that this form of populism combined with an accelerated but by no means new post-truth political environment, as seen in the United Kingdom with the Vote Leave campaigns infamous claim that the National Health Service (NHS) would have an extra £350 million per week post-Brexit, distorts the development of evidence based healthcare. In addition, by its focus on nativist public policy and reductions in entitlement among certain groups such as those proposed by the 2017 American Healthcare Act, right-wing populism risks excluding certain social groups from access to equitable healthcare thereby exacerbating health inequalities which in the United Kingdom context means changing the complexion of free at the point of access healthcare. One solution to this is to meaningfully engage with citizens to increase participation and democratic decision-making in healthcare policy and implementation. Speed and Mannion note that the populist right attack on experts focuses upon an ‘expertocratic’ professional elite and often emphasises strong, charismatic leadership as one antidote to a managerial class as exemplified by Donald Trump in the United States. However, if we were to take a broader view of populism to include a left wing populism then it is possible to envisage a role for this form of political engagement in developing more inclusive healthcare policy. Laclau’s nuanced definition of populism makes room for demands put forward by distinct displaced groups who make demands across a series of equivalential chains against the elite.^2^ In this form of populism a democratic engagement can occur whereby institutional forms of politics are rejected in favour of ‘the people’ taking greater control over the decisions that affect their lives.^2^ While there is potential for this form of populism to increase participation in healthcare policy, citizen engagement initiatives often merely offer further cover for market-based reform that arguably leads to less democratic participation and accountability.^3^ It is important to examine the complexities of participation in general and patient and public involvement (PPI) in particular in order to point to a route away from right-wing populism towards meaningful citizen participation in healthcare policy decision-making. The authors point to the development of “new technological services such as crowdsourcing, ‘open source’ systems and Bazaar forms of citizen governance.”^1^ However while these models of software based, online and transaction based economics^4^ may have potential to enhance some forms of citizen participation, the risk is that focusing on one technocratic approach will recreate a “professional ‘expertocratic’ political class”^1^ that Speed and Mannion identify as a partial cause of the democratic deficit in healthcare policy. It is therefore necessary to also consider other forms of citizen participation in healthcare that can envisage a form of populist engagement with marginalized groups of citizens who are at risk of political exploitation by populist leaders and those groups who are at greatest risk of exclusion by nativist health policies.

In considering citizen participation as a goal in healthcare policy and distinguishing it from the ‘fake news’ message of ‘taking back control’^5^ that was promised to UK citizens by the Leave EU campaign in relation to the NHS and other public institutions, it is necessary to first interrogate the construct of participation itself, a construct like populism that is vague and slippery to define. In contemporary discussions of participation in healthcare, one variety of which is referred to as PPI in the United Kingdom, the tension between consumer choice and citizen voice models of engagement are often centre stage.^6^ By focusing on a consumer choice model, PPI bodies such as Healthwatch operate in a space where, according to Carter and Martin, “distinctions between the market, the state and civil society have become increasingly blurred.”^6^ and the role of democratic representation becomes subsumed under the need to represent an increasingly commodified form of ‘patient experience.’^7^ Bodies such as Healthwatch can have their independence compromised by some local branches tendering for commissions and operating as for profit interest companies.^5^ The constitution of Healthwatch under the 2012 Health and Social Care Act represents its configuration as a top down, ‘institutional politics’ as opposed to populist^2^ solution to PPI. This instituional form of PPI can be seen in Healthwatch’s tendency to promote “choice rather than voice,”^6^ thus emphasizing consumer as opposed to inclusive populist forms of participation. This may undermine its capacity to advocate and campaign for targeted marginalized groups such as left behind indigenous groups likely to be seduced by right wing populism and also the immigrant groups discriminated against and suffering from health inequalities as a result of nativist health policy. The overarching risk with top down approaches to participation in an accelerated post-truth healthcare arena is that they recreate the sorts of democratic deficit that gave rise to right wing populist healthcare policies in the first place.^8^ Therefore, it is important to also consider bottom up approaches to participation that can fulfill Speed and Mannion’s suggestion for citizen engagement in health policy to alleviate the risk of nativist populism hijacking the agenda through manipulation of some groups and the exclusion of others.

## Community Psychology as a Process of Participation in Healthcare Policy and Implementation

One approach to bottom up participation in healthcare policy that draws on and develops preexisting capacity among populations^9^ is the field of Community Psychology. Community psychology can be defined briefly and narrowly, as an approach to the application of psychological knowledge that “emphasizes a level of analysis and intervention beyond the individual and his or her immediate interpersonal settings.”^9^ In this way it engages with the health of populations at a wider systemic level, avoiding the “cult of the immediacy”^10^ that bedevils much psychological theory and practice, and is interested in intervention at a variety of levels from micro through macro.^11^ In addition, community psychology “offers a framework for working with those marginalized by the social system that leads to self-aware social change with an emphasis on value based, participatory work, and the forging of alliances.”^12^ Community psychology internationally has developed public health interventions to reduce health inequalities with a number of marginalized groups, including; fathers of young children^13^; women living in a socially deprived community^14^; refugees, asylum seekers and migrant workers with mental health problems^15^; and LGBT (lesbian, gay, bisexual, and transgender) communities.^16^ What unifies these disparate approaches, in a sense similar to a chain of equivalences^2^ that can unite disparate groups in a populist left agenda, with diverse populations is their action research grounded, bottom up, strengths based approach to working alongside, as opposed to on or for, marginalized groups in order to facilitate second order systemic change.^17^ They also develop models of participation that eschew a consumerist approach, and instead prioritise citizen participation in decision-making, and an increase in democratic accountability. A further contribution that community psychology approaches can make to Speed and Mannion’s vision of “robust processes of democratic engagement”^1^ in healthcare policy and implementation is the field’s use of the Freirian theory of conscientisation^18^ as a key outcome goal in intervention. Conscientisation refers to a process whereby “people become aware of the political, socioeconomic and cultural contradictions that interact in a hegemonic way to diminish their lives.”^19^ Enabling a process of conscientisation to develop through participation in health policy would place require all actors; community psychologists, community members and other stakeholders to think critically about the social structures and processes that maintain or exacerbate health inequalities. Given that a key plank in the appeal of right wing populism is that it offers simplistic solutions to complex social problems through the development of narratives about ‘the other,’ analysis of wider social influences on health policy and inequalities, such as the privitisation agenda currently operating in the United Kingdom, has the potential to disrupt these populist agendas in small scale ways. Engagement in participatory practices such as those exemplified by the field of community psychology would also offer the possibility that populations, particularly smaller scale communities, can develop expertise in areas of health policy that most directly affect them, thereby contesting the populist notion that expertise only resides among an unaccountable elite. While the field of community psychology offers the possibility of engagement with marginalized groups to increase community level awareness of health policy and health inequalities, including crucially the social processes that might underpin them, it is unable to singlehandedly provoke the large-scale changes necessary to impact upon populist agendas, or to develop a different form of inclusive populism that can bring groups together in a chain of equivalences rather than exclude some on the basis of identity or other differences. However, through engagement with top down approaches such as Healthwatch, there is the possibility that small scale community psychology initiatives like those listed above can, through galvanizing groups of people as opposed to individuals, enable local communities to engage with participatory health policy discussions without immediately being coopted by established interests.^20^ It also holds out the possibility that through engagement with health policy and implementation in areas of specific concern, people can engage primarily as citizens, thereby avoiding falling into the ‘consumer trap’ that can limit the scope of PPI, and reduce the health of populations to a series of individual transactions and balance sheets, rather than a reflection of wider governmental accountability and a key plank of a society’s social contract.

## Ethical issues

Not applicable.

## Competing interests

Author declares that he has no competing interests.

## Author’s contribution

DT is the single author of the paper.
